# Critical Assessment of Techniques for the Description of the Phase Composition of Advanced High-Strength Steels

**DOI:** 10.3390/ma12244033

**Published:** 2019-12-04

**Authors:** Anna Knaislová, Darya Rudomilova, Pavel Novák, Tomáš Prošek, Alena Michalcová, Přemysl Beran

**Affiliations:** 1 Department of Metals and Corrosion Engineering, University of Chemistry and Technology, Technická 5, 166 28 Prague, Czech Republic; darya.rudomilova@vscht.cz (D.R.); panovak@vscht.cz (P.N.); prosekt@vscht.cz (T.P.); michalca@vscht.cz (A.M.); 2 Nuclear Physics Institute, Czech Academy of Sciences, 250 68 Řež, Czech Republic; pberan@ujf.cas.cz; 3 European Spallation Source ERIC, 222 70 Lund, Sweden

**Keywords:** high strength steels, microstructure, etching agents

## Abstract

The phase composition and portion of individual phases in advanced high-strength steels (AHSS) CP1000 and DP1000 was studied by complementary microscopic and diffraction techniques. CP1000 and DP1000 steel grades have a high strength-to-density ratio and they are used in many applications in the automotive industry. The microstructure of the CP1000 “complex phase” steel consists of ferrite, bainite, martensite and a small amount of retained austenite. DP1000 is a dual phase steel, which has a structure of a ferritic matrix with islands of martensite and a minor amount of retained austenite. The influence of selected etchants (Nital, LePera, Beraha I, Nital followed by metabisulfite, Nital followed by LePera, and Nital followed by Beraha I) on the microstructure image is described. X-ray diffraction, neutron diffraction and light optical, scanning and transmission electron microscopy were used in this work for advanced characterization of the microstructure and phase composition. The information provided by each technique is critically compared.

## 1. Introduction

Over the last decades, the automotive industry has searched for new materials with improved mechanical properties in combination with low density. The aim is to decrease the weight of components and CO_2_ emissions, to improve crash safety of automobiles and to reduce gasoline consumption [1,2,3,4,5,6]. Materials used in the automobile industry should be easily formable, weldable, coatable and repairable [7,8,9]. Advanced high-strength steels (AHSS) offer higher strength and better formability compared to conventional steels, which is given by improved phase composition [1,10,11,12]. Their tensile strength ranges from 500 to 1600 MPa, which makes them attractive for application in various car components [13,14]. The strength is controlled by the carbon content. The addition of alloying elements in small quantities (microalloying) is important for refinement of the microstructure and strengthening the ferrite phase, especially in dual-phase steels [15].

Complex phase steel (CP, ferritic-martensitic-bainitic), described in this work, meets the above-mentioned requirements and can be used for innovative lightweight automotive structural parts, reaching a yield strength of approximately 800 MPa, an ultimate tensile strength around 1000 MPa and ductility (elongation at break) of at least 7% [16]. This kind of steel finds application in door impact bars, side impact beams, seat mounting rails, bumper brackets, stiffeners, sills or parts of the chassis components [3]. CP steel is composed of phases with a lower hardness difference like martensite, tempered martensite and bainite. A small amount of retained austenite is also present [17,18]. The fine microstructure of high-strength phases (martensite, bainite) increases yield strength [3,19,20]. Dual-phase steel (DP, ferritic-martensitic) is characterized by low yield strength, high work hardening, high tensile strength, continuous yielding [21], as well as high uniform and fracture elongation due to the martensite phase islands present in the microstructure [22,23,24,25]. DP steel consists of a soft ferrite matrix with finely dispersed martensite islands [15,26,27,28,29]. Martensite provides high strength while ferrite is responsible for plasticity [23,30,31]. In general, the microstructure is very fine and combines both deep-drawing capacity and high strength. A lower amount of martensite may result in deficient free dislocations and insufficient tensile strength and high yield strength [22,23]. A minor amount of retained austenite is present in DP steel if carbon-enriched austenite does not transform entirely to martensite. The small quantity of retained austenite does not significantly change the specific properties of this steel [22]. DP steel is ideal for applications in complex structural components for lightweight vehicles, such as car body panels, wheels and bumpers [23,32].

Regarding the characterization of microstructure and phase composition of steel, there are many techniques available, comprising diffraction methods (X-ray, electron and neutron diffraction or EBSD) [33], metallography using optical [34,35] and scanning electron microscopy, and local chemical analysis methods (EDS and WDS methods). The identification of microstructural constituents in medium- and high-carbon steel is relatively simple due to high difference in carbon content, also causing a measurable difference in the lattice parameters. Due to tetragonality of martensite, this metastable phase could be easily recognized from ferrite or bainite [33]. For AHSSs with low carbon content, recognition of these phases is much more problematic, being based mostly on experience with visual determination of the phases in optical micrographs. Therefore, the aim of this work was to determine phase compositions of CP and DP AHSSs and compare available metallographic and diffraction techniques in view of their applicability for these materials.

## 2. Materials and Methods

CP steel with ultimate tensile strength (UTS) of about 960 MPa (CP1000) and DP steel with the same UTS (DP1000) were provided by voestalpine Stahl (Linz, Austria). Their typical chemical composition is given in Table 1. 

Specimens cut from rolled sheets with thickness of 0.8 mm were embedded in epoxy resin, ground and polished by diamond paste and etched by selected agents. An overview of used etching agents is shown in Table 2. In addition to etchants listed in Table 2, combinations Nital followed by MBS, Nital followed by LePera and Nital followed by Beraha were tested. Material microstructure was studied by light optical microscopy (Olympus DSX510, Tokyo, Japan), scanning electron microscopy (SEM; ZEISS EVO 15, Oberkochen, Germany) and transmission electron microscopy (TEM; JEOL 2200FS, Akishima, Japan). TEM samples were prepared by ion polishing using Gatan PIPs (precision ion polishing machine) (Gatan, Pleasanton, CA, USA).

Phase composition of CP1000 and DP1000 was determined by X-ray diffraction analysis (XRD) and neutron diffraction. XRD was performed using a Panalytical XpertPro X-ray diffractometer (PANalytical, Almelo, Netherlands).

Neutron diffraction measurements of CP1000 and DP1000 at room temperature were performed on the instrument MEREDIT@NPI (ÚJF, Řež, Czech Republic). Diffraction patterns were collected between 4° and 144° of 2θ with a step of 0.08° using a neutron wavelength of 1.46 Å. Thanks to the usage of mosaic copper monochromator on reflection 220, a small λ/2 (0.73 Å) contamination of 0.4% in the incoming neutron beam was considered during refinement. Five flat sample sheets with a cross-section of 20 × 20 mm^2^ and a thickness of 1 mm were stacked together and placed in the neutron beam. The sample stacks were rotating along the vertical axis perpendicular to the sheet surface to minimize the influence of the texture effect on the diffracted intensities. Data analysis was performed by the full pattern fitting using FullProf software (Version 6.30 -Sep2018) [36]. To properly extract the microstructural and structural information, the profile function obtained by measurement of SiO_2_ standard sample at the same conditions was used.

## 3. Results and Discussion

### 3.1. Diffraction Analysis

**Figure 1:**
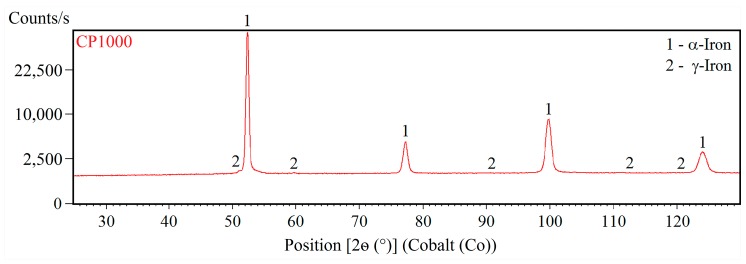
XRD pattern of CP1000.

**Figure 2:**
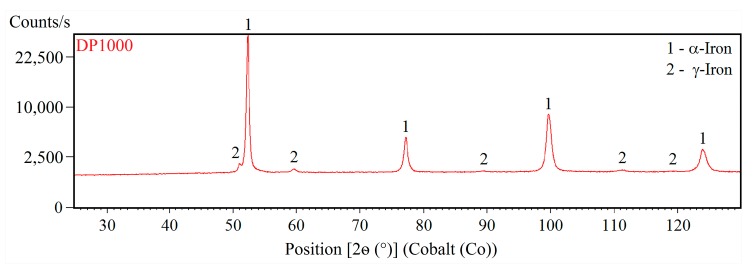
XRD pattern of DP1000.

Figure 1 and Figure 2 shows XRD patterns of CP1000 and DP1000. X-ray diffraction distinguishes various crystallographic modifications of iron (α-Fe, γ-Fe). However, it is unable to differentiate ferrite from martensite and bainite. Due to the low content of carbon in the investigated steels it can be expected that the martensite would have nearly cubic structure, as well as ferrite. Alpha (α-Fe, bcc) and gamma (γ-Fe, fcc) iron phases were quantified by the Rietveld method in order to refine the lattice constants and peak shapes. CP1000 contains 98.8 wt.% of α-Fe (ferrite, martensite and bainite) and 1.2 wt.% of γ-Fe (retained austenite). DP1000 contains a higher quantity of 2.6 wt.% γ-Fe and the content of α-Fe, i.e., martensite and ferrite, reaches 97.4 wt.%.

**Figure 3:**
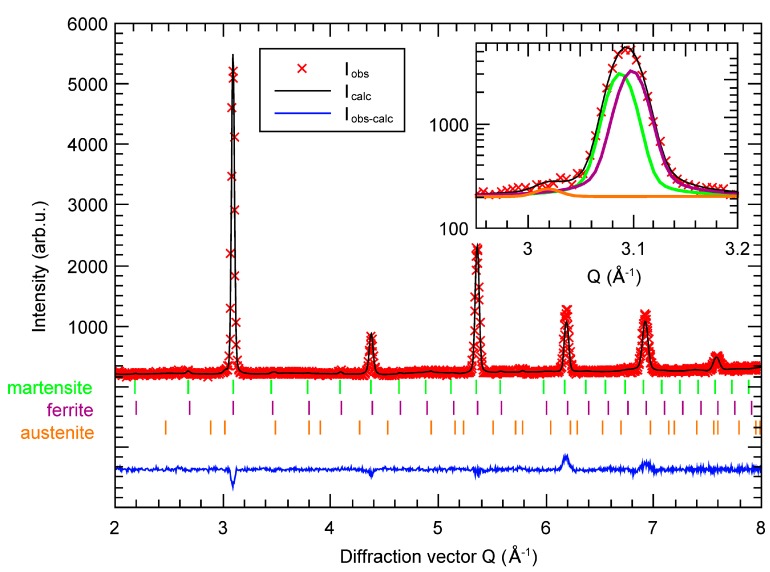
Measured (I_obs_) and calculated (I_calc_) neutron diffraction pattern of CP1000. The difference (I_obs_–I_calc_) and Bragg reflection for each considered phase are also shown. The inset shows the zoomed area of the first reflection and the contribution of each phase to the profile.

**Figure 4:**
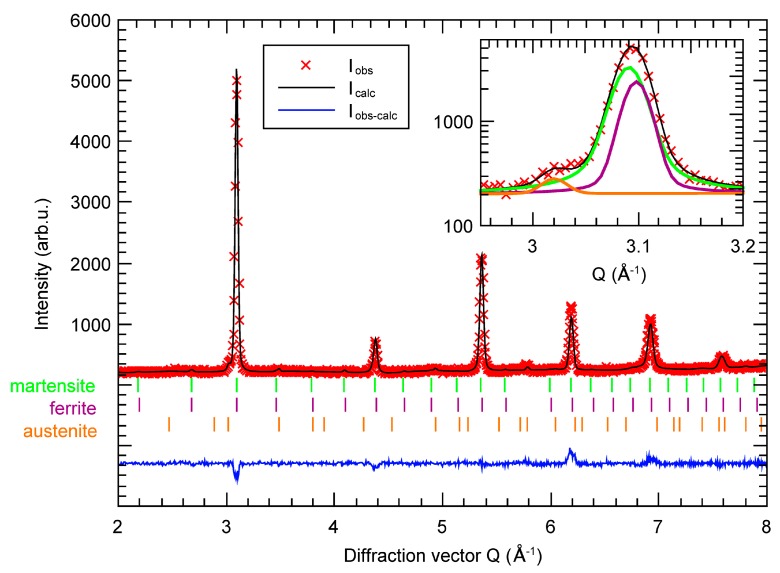
Measured (I_obs_) and calculated (I_calc_) neutron diffraction pattern of DP1000. The difference (I_obs_–I_calc_) and Bragg reflection for each considered phase are also shown. The inset shows the zoomed area of the first reflection and the contribution of each phase to the profile.

In order to analyse the phase composition of tested AHSS across the whole sample volume and, hence, more reliably, the neutron diffraction has been used. Measured and calculated neutron diffraction patterns for both samples are shown in Figure 3 and Figure 4. The neutron diffractograms are very similar to the X-ray ones and reflections of α-Fe (ferrite, martensite) and γ-Fe (retained austenite) are clearly visible. However, the reflection of α-Fe are slightly asymmetric and far broader than the standard instrument profile function. The broadening can indicate micro-strain within the phase but the shape of asymmetry matches with a two-reflections-overlap shape. Tetragonality of martensite is supposed to be very low due to the low concentration of carbon and thus the structure was fitted by a cubic one during the refinement. The tiny carbides in the bainite phase were not visible on the diffraction pattern of the CP1000 sample and the bainite phase was considered as ferrite. The shape of the reflection was well reproduced and it reveals a 0.3% larger cell for the martensite phase. The lattice parameter for ferrite, martensite and retained austenite was 2.866 Å, 2.876 Å and 3.605 Å, respectively. Refined phase fractions of ferrite 53% ± 1% and martensite 46% ± 1% are in agreement with results of metallographic analyses (see below). The content of retained austenite of 0.9% ± 1.0 % is slightly lower than the value determined by XRD, probably due to the larger tested volume in neutron diffraction. There can also be local differences of the phase ratio depending on the distance from surface due to quenching.

The analysis was done identically for DP1000. The martensite unit cell was found to be 0.2% larger in comparison with the ferrite one. The lattice parameter for ferrite, martensite and retained austenite was 2.866 Å, 2.874 Å and 3.602 Å, respectively. The refined phase fractions are as follows: Martensite 63% ± 2%, ferrite 35% ± 2% and retained austenite 2.1% ± 1.0%. The results are in good agreement with other analyses shown below.

Further microstructural analysis revealed a high level of micro-strain and texture in both martensite and ferrite. A significant increase of the intensities of {110} reflections indicates preferred orientation of those planes in perpendicular direction to the sample sheet. The level of micro-strain of the martensite phase in CP1000 is three times higher than in DP1000. The ferrite phase shows only subtle differences.

### 3.2. Electron Microscopy Microstructure Analysis

**Figure 5:**
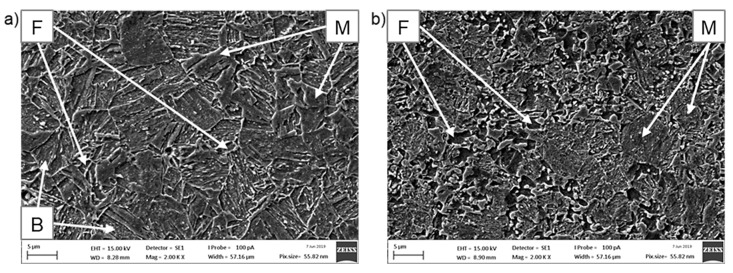
Microstructure of AHSSs from SEM after etching with Nital: (**a**) CP1000, (**b**) DP1000; F—ferrite, M—martensite, B—bainite.

Figure 5 shows SEM images of CP1000 and DP1000 microstructures etched by Nital. Individual phases were identified based on comparison with available literature [37,38,39,40,41,42]. The phase composition was evaluated by image analysis. In CP1000, there is approximately 48% of martensite, 32% of ferrite and 20% of bainite. DP1000 is composed of 64% of martensite and 36% of ferrite. These results correspond well to the results from neutron diffraction.

**Figure 6:**
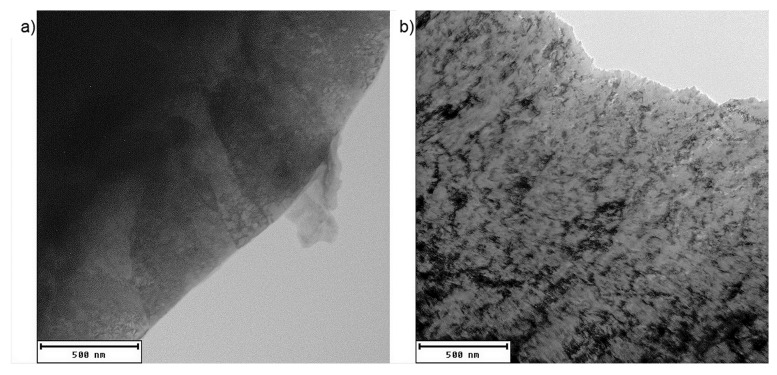
Microstructure of (**a**) CP1000 and (**b**) DP1000 observed by TEM.

**Figure 7:**
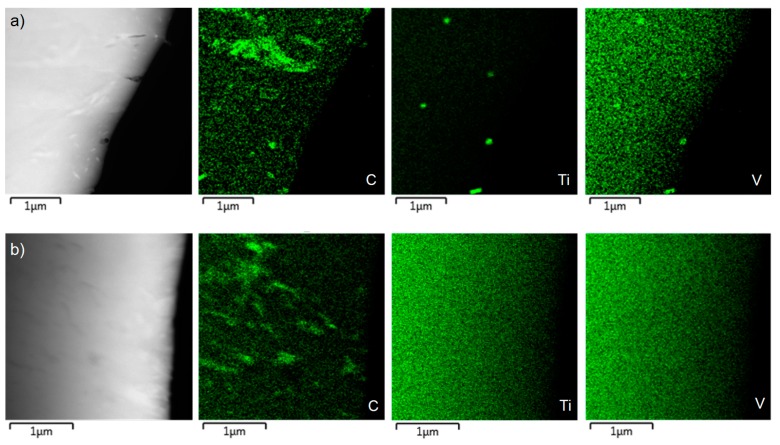
Maps of distribution of individual elements (TEM-EDS) in: (**a**) CP1000 steel, (**b**) DP1000 steel.

TEM observation was complicated by the heterogeneity of studied materials. As the field of view in TEM sample is very small, internal structure of a single random grain is obtained. TEM observation did not help in recognizing the individual phases (Figure 6). Due to the low carbon content and, hence, similar crystal structure, the difference between bainite and martensite is not well distinguishable. The micrograph in Figure 6b shows areas with high dislocation density (black), probably in soft ferrite phase close to the interface with hard martensite and/or bainite. Carbides in CP1000 contain titanium and vanadium, i.e., microalloying additives (see the energy dispersive spectroscopy (EDS) map in Figure 7). The size of the carbides is 0.1–0.2 µm. No carbides of titanium and vanadium were distinguished in DP1000.

### 3.3. Light Optical Microscopy Microstructure Analysis 

**Figure 8:**
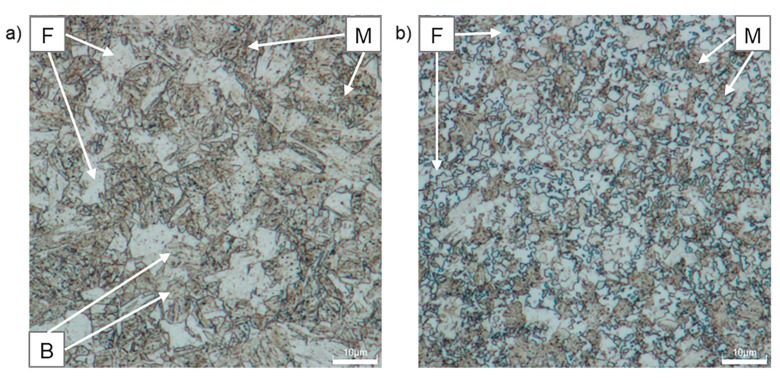
Optical micrographs of AHSSs after etching with Nital: (**a**) CP1000, (**b**) DP1000; F—ferrite, M—martensite, B—bainite.

Figure 8 shows the microstructure of CP1000 and DP1000 etched by Nital. The etchant makes the grain boundaries of ferrite visible. Nital etches the ferrite phase much slower and less intensely than the martensite phase, thus, the morphology and area fraction can be relatively easily determined with this technique [43]. Ferrite is less etched and appears light, whereas martensite is darker. Globular or plate martensite forms individual islands in CP1000 and continuous network in DP1000. From these images, amounts of individual phases have been estimated. CP1000 contains 46% martensite, 29% ferrite and 25% bainite, while DP1000 is formed by 65% martensite and 35% ferrite. Retained austenite is not distinguishable in the light optical microscope images. These results correspond well with SEM and neutron diffraction data.

**Figure 9:**
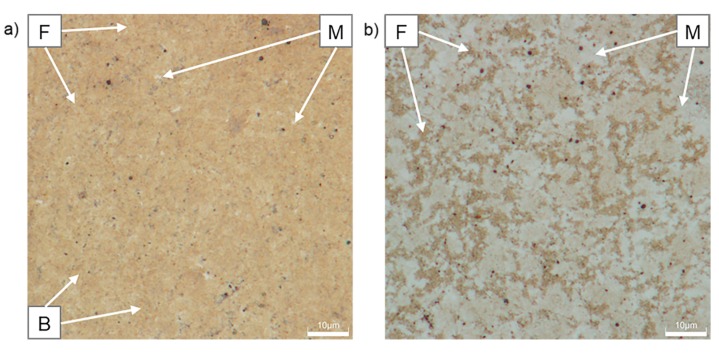
Optical micrographs of AHSSs after etching with LePera: (**a**) CP1000, (**b**) DP1000; F—ferrite, M—martensite, B—bainite.

Figure 9 shows the microstructure of CP1000 and DP1000 etched with LePera etching agent. This etchant is usually used to determine the amount of martensite. LePera etching reveals ferrite in dark brown and bainite and tempered martensite in light brown, and austenite/martensite phases appear as small white units [44]. This etchant is not suitable for CP1000 steel since it is impossible to recognize individual phases. The application of this etchant for DP1000 enables distinguishing ferrite (brown colour) from martensite and tempered martensite (white, light brown colour) [45]. 

**Figure 10:**
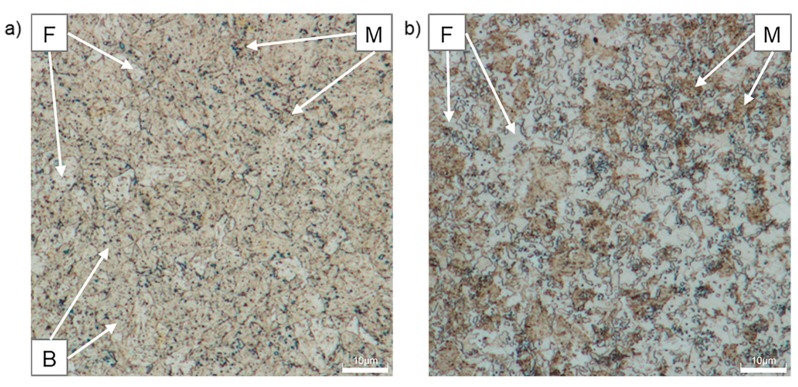
Optical micrographs of AHSSs after etching with Beraha: (**a**) CP1000, (**b**) DP1000; F—ferrite, M—martensite, B—bainite.

Figure 10 shows the microstructure of CP1000 and DP1000 etched with Beraha etchant. The lighter phase corresponds to ferrite, while the darker phase corresponds to martensite. Bainite in CP1000 and tempered martensite in DP1000 is coloured light brown [45].

**Figure 11:**
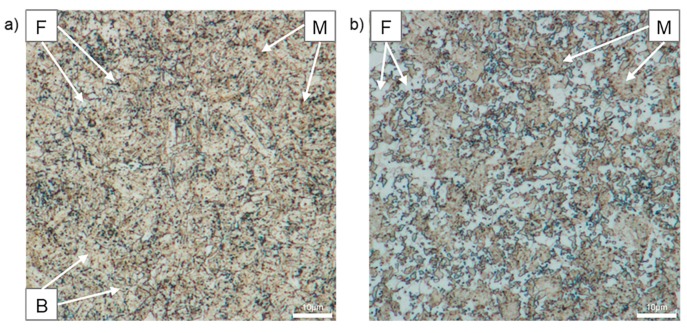
Optical micrographs of AHSS after etching with Nital followed by MBS; (**a**) CP1000, (**b**) DP1000; F—ferrite, M—martensite, B—bainite.

Figure 11 shows the microstructure of CP1000 and DP1000 etched with Nital followed with sodium metabisulfite, Na_2_S_2_O_5_ (MBS). This etching provides a high contrast of the grain boundaries because of the combination of a grain boundary etching with a surface layer etching. By a combination of Nital and MBS, tempered martensite and bainite both appear brown in the ferrite matrix (white coloured) [45].

**Figure 12:**
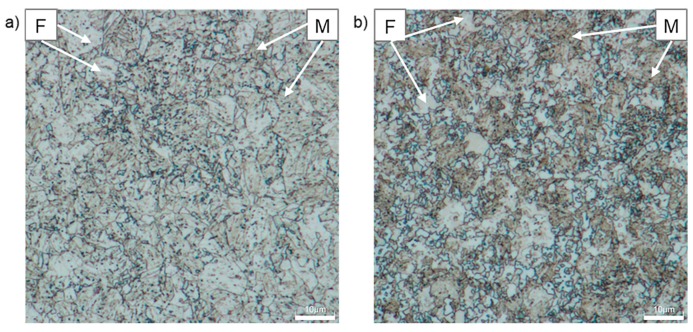
Optical micrographs of AHSS after etching with Nital followed by LePera: (**a**) CP1000, (**b**) DP1000; F—ferrite, M—martensite, B—bainite.

Figure 12 shows the microstructure of CP1000 and DP1000 after etching with Nital followed by LePera. Nital makes the ferrite grain boundaries visible and then LePera had no effect on the distinction of selected phases. The ferrite phase is white (less etched) and the martensite is brown (dark).

**Figure 13:**
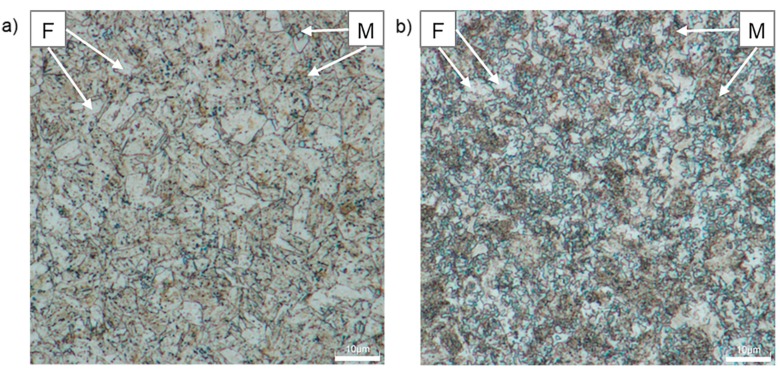
Optical micrographs of AHSS after etching with Nital followed by Beraha: (**a**) CP1000, (**b**) DP1000; F—ferrite, M—martensite, B—bainite.

Finally, Nital followed by Beraha was used for etching (Figure 13). The microstructure with visible grain boundaries of ferrite after etching with Nital stayed the same after etching with Beraha. Application of Beraha etchant after Nital also had no effect on the contrast of individual phases in CP1000 and DP1000.

Table 3 shows the summary results of phase composition of CP1000 and DP1000 obtained by light optical microscopy after application of different etchants. Sophisticated etching agents did not show better results than etching with Nital. The bainite present in CP1000 was only observed after etching with Nital. By etching with Beraha I, it was possible to distinguish martensite and bainite from ferrite. Other etching agents were not suitable for recognition of individual phases in CP1000. Phase identification in dual-phase steel was easier. Portions of the present phases after etching with all etchants were calculated, resulting in the amount of martensite ranging between 61% and 70% and the amount of ferrite between 30% and 39%. The combination of Nital with other etching agents did not bring any additional information.

### 3.4. Summary

Table 4 shows the summary results of the phase composition of CP1000 and DP1000 obtained from selected microscopic and diffraction techniques. First, the steels were subjected to X-ray diffraction, which was unable to distinguish ferrite from martensite and bainite due to low carbon content and, hence, the same crystallographic structure. The amount of retained austenite was determined by Rietveld refinement of the XRD patterns. Neutron diffraction enabled distinguishing the martensite from ferrite based on small differences in the lattice parameters, but the amount of bainite was impossible to quantify because its main constituent is ferrite. The amount of retained austenite detected by XRD was slightly higher than for nuclear diffraction, probably due to local variations in the microstructure. 

Due to very high resolution, TEM is useful for the determination of the presence and distribution of fine carbides of microalloying elements. It is not a practical tool for phase identification and quantification.

**Figure 14:**
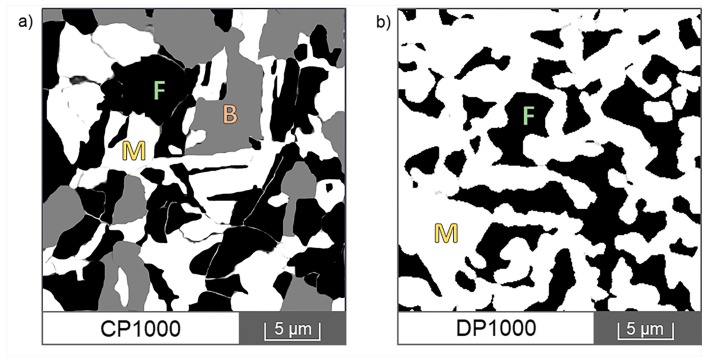
Schematic microstructure of: (**a**) CP1000 and (**b**) DP1000; F—ferrite, M—martensite, B—bainite.

Simple light optical microscopy and SEM metallographic techniques enabled phase identification except for the presence of retained austenite. This is because the retained austenite is present in a form of very small particles between martensite laths and, hence, it is not recognizable by these techniques [46,47,48]. Image analysis of microstructures from SEM and light optical microscopy shows similar results, which corresponds to the results from neutron diffraction. A schematic of the microstructure of the two studied steels is illustrated in Figure 14. 

## 4. Conclusions

Identification and quantification of the phases in CP1000 and DP1000 advanced high-strength steels was achieved by combining data from several complementary techniques:

- X-ray diffraction was the only technique able to determine the amount of retained austenite, but it was unable to make a distinction between ferrite, martensite and bainite. The reason was that due to similarly low carbon content, he lattice parameters were also similar and below the resolution of this technique.
- Neutron diffraction distinguished martensite from ferrite based on small differences in lattice parameters. The amount of bainite could not be quantified because it is not possible to detect cementite in bainite by neutron diffraction.
- The main contribution of transmission electron microscopy was its ability to detect and show the distribution of fine carbides in the structure. Phase composition was better described by other techniques.
- Scanning electron and light optical microscopy on specimens etched with Nital provided the most comprehensive results, being able to distinguish and quantify ferrite, martensite and bainite phases. Subsequent etching with other solutions did not bring any improvement.

For complete information about the microstructure constituents and their fractions, at least two techniques need to be combined. XRD, SEM and light optical microscopy after Nital etching are considered optimal for microstructure characterization of AHSS steels.

## Figures and Tables

**Table 1:** Chemical composition of CP1000 and DP1000.

Element (wt.%)	C	Si	Mn	Cr + Mo	Nb + Ti	V	P	S	B	Al	Fe
	Max.	Max.	Max.	Max.	Max.	Max.	Max.	Max.	Max.	Total	
CP1000	0.23	1.00	2.70	1.00	0.15	0.20	0.08	0.02	0.01	0.015–2	Bal.
DP1000	0.18	0.80	2.50	1.40	0.15	0.20	0.08	0.02	0.01	0.015–1.4	Bal.

**Table 2:** Overview of used etching agents.

Etching Agent	Composition	Etching Principle
Nital	5 mL HNO_3_, concentrated 95 mL C_2_H_5_OH (ethanol)	Grain boundary etching
LePera	50 mL Na_2_S_2_O_5_, 1% aqueous solution 50 mL (O_2_N)_3_C_6_H_2_OH (picric acid), 4% ethanol solution	Uniform surface layer etching
Beraha I	Basic solution: 24 g NH_4_FHF (ammonium bifluoride) 200 mL HCl 1000 mL H_2_O 100 mL of basic solution 1 g K_2_S_2_O_5_ (potassium disulfite)	Uniform surface layer etching
Metabisulfite (MBS)	Na_2_S_2_O_5_ (sodium metabisulfite), 10% aqueous solution	Uniform surface layer etching

**Table 3:** Phase composition of CP1000 and DP1000 obtained by light optical microscopy; vol.%.

Etching Agent	Portion of Phases		Comments
	CP1000	DP1000	
Nital	46% M 29% F 25% B	65% M 35% F	Ferrite grain boundaries visible, martensite phase revealed
LePera	Individual phases were not recognized	70% M 30% F	Determination of the amount of martensite in DP1000, not suitable for CP1000
Beraha I	75% M + B 25% F	65% M 35% F	Impossible to distinguish bainite and martensite in CP1000
Nital followed by MBS	Impossible to recognize individual phases	61% M 39% F	High contrast of grain boundaries in DP1000, not suitable for CP1000
Nital followed by LePera	Impossible to recognize individual phases	64% M 36% F	Ferrite grain boundaries visible, not suitable for CP1000
Nital followed by Beraha I	Impossible to recognize individual phases	63% M 37% F	Ferrite grain boundaries visible, not suitable for CP1000

Note: F—ferrite, M—martensite, B—bainite.

**Table 4:** Phase composition of CP1000 and DP1000 steel sheets obtained by compared techniques; vol.%.

Method	CP1000	DP1000
XRD	98.8% F + M + B 1.2% A	97.4% F + M 2.6% A
Neutron diffraction	46% M 53% F + B 1% A	63% M 35% F 2% A
SEM	48% M 32% F 20% B	66% M 36% F
Light optical microscopy (Nital)	46% M 29% F 25% B	65% M 35% F

Note: F—ferrite, M—martensite, B—bainite, A—austenite.
